# Diversity of toxic and phytopathogenic *Fusarium* species occurring on cereals grown in Karnataka state, India

**DOI:** 10.1007/s13205-016-0399-5

**Published:** 2016-02-13

**Authors:** H. Nagaraja, G. Chennappa, K. Poorna Chandra Rao, G. Mahadev Prasad, M. Y. Sreenivasa

**Affiliations:** Department of Studies in Microbiology, University of Mysore, Manasagangotri, Mysore, 570 006 Karnataka India

**Keywords:** Cereals, Mycotoxins, Fungal spoilage, *Fusarium*

## Abstract

A total of 198 cereal samples (53 maize, 54 sorghum, 37 paddy and 54 wheat) were collected from 11 districts of Karnataka to understand the percent infection (PI), relative density (RD) and their frequency (Fr) caused by *Fusarium* spp. All samples were screened by agar plating method using MGA 2.5 agar media and incubated at 25 ± 2 °C for 3–5 days. The study revealed the association of 10 different *Fusarium* species known trichothecene producers such as *Fusarium acuminatum*, *F. avenaceum*, *F. crookwellense*, *F. culmorum*, *F. equiseti*, *F. graminearum*, *F. nivale*, *F. poae*, *F. sambucinum and F. sporotrichioides* along with non-trichothecene producers like *F. anthophilum*, *F. oxysporaum*, *F. proliferatum*, *F. semitectum*, *F. solani*, and *F. verticillioides*. All the ten isolated potential trichothecene producing *Fusarium* species were analyzed for their ability to produce trichothecenes by using thin layer chromatography method. The highest infection of *Fusarium* spp. in maize was by *F. verticillioides* with PI of (2.95 %), with RD of (15.16 %) and highest Fr was by *F. graminearum* (79.24 %) and the lowest was *F. avenasium* with PI of (0.13 %). For sorghum maximum PI was by *F. verticillioides* (3.02 %), with *F. graminearum* having highest RD (14.39 %) and with *F. verticillioides* highest Fr. (72.22 %). In paddy highest PI was by *F. verticillioides* (3.21 %) and the least was by *F. avenaceum* (0.09 %). Similarly in wheat the highest PI was by *F. verticillioides* (2.76 %) while lowest was by *F. avenaceum* (0.10 %). The highest Fr was with *F. graminearum* (79.62 %) while the lowest was by *F. avenaceum* (3.70 %) and the highest RD was by *F. graminearum* (22.04 %) and lowest was by *F. solani* (0.72 %). The manually identified *Fusarium* spp. were further confirmed by PCR-based detection using ITS1 and ITS4 primers followed by sequencing of the PCR amplicons. PCR studies confirmed that all the tested fungal isolates belongs to *Fusarium* spp. with the amplicon size of 600 bp. Sequencing and the blast data from NCBI data base confirmed the sequence similarity of 99 % to the genus *Fusarium* and accession numbers were obtained. Chemotyping studies showed that the isolated *Fusarium* species are known to produce different types of trichothecenes. The study revealed the diversity in phytopathogenic *Fusarium* spp. in major cereal crops growing in different agro-climatic regions of Karnataka, India.

## Introduction

Cereals are mainly cultivated for food, feed and fodder. These are grown in greater quantities worldwide than any other crop to provide food energy as staple crops. The cereal grains such as maize, sorghum, wheat, rye, barely and paddy are commonly contaminated with fungi. Fungi are one of the major contaminants of food and feed grains causing biodeterioration. These grains are highly nutritious and as such they are prone to get contaminated at any point of time from harvest to storage. A wide range of fungal species including *Aspergillus*, *Fusarium*, *Penicillium*, *Alternaria*, *Cleviceps*, *Monascum*, *Cephalosporium*, *Drechslaria*, *Nigrospora*, *Trichoderma* have been reported to infect to cereal grains (Bhattacharya and Raha 2002).

The genus *Fusarium* is one of the most important fungal species occurring worldwide and is chiefly associated with cereal crops. *Fusarium* species are seed-borne, seed-transmitted, soil-borne and soil-transient plant pathogens. They cause death of seedlings, seed abortion, kernel and seedlings rots, blight, chlorosis, vascular wilt, dieback, stunt and reduction in growth in a variety of host plants. Seed-borne characteristic of *Fusarium* species have been well documented on various crops including cereals (Bottalico 1998), some oil seeds (Geetha and Reddy 1990), sunflower (Kaur et al. 1990), linseed (Fitt et al. 1991) and many others.

*Fusarium* species are known to produce more than hundred secondary metabolites such as mycotoxins in which majority of them can unfavorably affect human and animal health. These toxins inhabit naturally in cereals and other agricultural foods and feeds, either individually or in specific clusters of two or more of them. The most common and important *Fusarium* mycotoxins frequently occurring at biologically significant concentrations in cereals are fumonisins, moniliformins and trichothecenes (Bottalico and Perrone 2002).

The reports on *Fusarium* contaminations in cereals in India are scanty and first outbreak of mycotoxin contamination was reported from Kashmir in 1987. In India, deoxynivalenol (DON) has been implicated along with some other mycotoxins in rice, sorghum and wheat. *Fusarium* mycotoxins were also reported from cereals such as maize, sorghum, wheat, barley, rice and with some feeds and food stuffs from Hyderabad region of Andhra Pradesh and Mysore region of Karnataka (Lincy et al. 2008). Incidence and diversity in *Fusarium* species associated with maize and sorghum samples collected from farm yards and local markets were also reported from different districts of Karnataka (Sreenivasa et al. 2011). In the present study emphasis was bestowed to understand the diversity, relative density, extent of infection and frequency of toxigenic *Fusarium* species occurring on a wide range of cereals grown as crops and from stored cereal grains.

## Materials and methods

### Collection of cereal samples

A total of 198 samples (53 maize, 54 sorghum, 37 paddy and 54 wheat samples) were collected from 11 districts of Karnataka, from agricultural field crops, local markets, APMC and co-operative yards. Approximately 1 kg of each sample was collected in a sterile zip lock polythene bag and stored at 4 °C in the laboratory until they are subjected for further mycological analysis.

### Mycological analysis

Information about percent infection (PI), relative density (RD) and percent frequency (Fr), were collected by placing cereal samples on selective media for the isolation of *Fusarium* species. Randomly selected 200 grains from each cereal were surface sterilized with 2 % sodium hypochlorite solution for 2–3 min, rinsed twice with sterile distilled water and seeds were blot dried. Samples were then placed on MGA 2.5 agar plates at the rate of 10 cereal grains per plate and incubated at 25 ± 2 °C for 5–7 days (Bragulat et al. 2004). The incubated plates were visualized for the fungal growth using stereo-binocular microscope and compound microscope. The representative isolates of different *Fusarium* species were transferred onto potato dextrose agar (PDA), to study the micro- and macro-morphological characteristics and identified up to the species level by using *Fusarium* identification keys and manual (Booth 1977; Leslie and Summerell 2006), and PI, RD and Fr of *Fusarium* species were recorded using the following formula described by Ghiasian et al. (2004).

$${\text{Percent infection}} = \frac{{{\text{Number of seeds/grains infected with }}Fusarium{\text{ species}}}}{\text{Total number of seeds/grains plated}} \times 100$$

$${\text{Frequency }}\left( \% \right) = \frac{{{\text{Number of samples infected with }}Fusarium{\text{ species}}}}{\text{Total number of samples analyzed}} \times 100$$

$${\text{Relative density }}\left( \% \right) = \frac{{{\text{Number of }}Fusarium{\text{ species isolated}}}}{{{\text{Total number of }}Fusarium{\text{ isolated}}}} \times 100$$

### Extraction of genomic DNA from *Fusarium* isolates

The extraction of *Fusarium* genomic DNA was performed as per the protocol described by Sreenivasa et al. (2008) with minor modifications. Each representative *Fusarium* isolate was freshly inoculated into potato dextrose broth in 2 mL microfuge tube and was incubated at 25 ± 2 °C for 4 days. The mycelium was centrifuged at 5000 rpm for 5 min at 4 °C. The supernatant was discarded and 500 µL lysis buffer (composition: Tris HCl 1.576 g in 10 mL of distilled water, pH 8; EDTA 3.722 g in 10 mL of distilled water; 8.3 mL 20 % SDS; PVP 1 g; 2 µL of 0.5 M lithium chloride in 10 mL distilled water. The working buffer composition: 1.3 mL of Tris HCL + 3.4 mL of EDTA + 10 mL of SDS from stock all in 10 mL distilled water) were added, ground with blunt ends of the disposable sterile micropipette tips and incubated on water bath at 65 °C for 15 min. Five hundred micro liter of phenol: chloroform (1:1) were added and gently vortexed for 1 min. The mixture was centrifuged at 3000 rpm at 4 °C for 5 min. The supernatant was transferred to a fresh 1.5 mL microfuge tube, equal volume of ice-cold iso-propyl alcohol was added and incubated at −20 °C overnight and centrifuged at 8000 rpm at 4 °C for 10 min. The supernatant was discarded and the resulting pellet was rinsed with 70 % ice-cold ethanol, centrifuged at 8000 rpm at 4 °C for 10 min and air dried at room temperature. Further re-suspended in 50 µL of nucleic acid free water and was used further for PCR quantification.

### PCR amplification

The extracted DNA was amplified using ITS-1 (5-TCC GTA GGT GAA CCT GCG G-3) and ITS-4 (5-TCC TCC GCT TAT TGA TAT GC-3) primers as described by Guo et al. (2004). The PCR conditions were: initial denaturation at 94 °C for 4 min, denaturation at 94 °C for 1 min, annealing at 55 °C for 1 min, extension at 72 °C for 1.5 min and final extension at 72 °C for 5 min.

### Sequencing and phylogenetic analysis of *Fusarium* species

The PCR amplicons of all *Fusarium* spp. were sequenced at Sci Genome, Cochin, India. After sequencing, all the sequences were confirmed with NCBI, BLAST database for the identity of the isolates based on previously published database sequences. Phylogenetic tree was constructed using Mega 5.0 online software of UPGMA Neighbor Joining method. The same sequence reads were deposited at NCBI and obtained accession numbers for each representative *Fusarium* sp.

### Mycotoxicological analysis of *Fusarium* species by TLC

Freshly prepared 500 mL of PDB was inoculated with each testing *Fusarium* sp. and incubated for 45 days at room temperature. The resulting broth was centrifuged at 8000 rpm for 8 min and the filtrate was extracted with acetonitrile–water (84:16). The extract was defatted with equal amount of hexane and further extracted with equal amount of dichloromethane after adding a pinch of Na_2_So_4_, kept at room temperature for 5 min to absorb the moisture. The extract was flash evaporated on water bath at 55 °C at 200 rpm. The crude extract was collected and dried in vacuum evaporator for 1 h at 55 °C. The dried sample was diluted with acetonitrile and used for chromatographic analysis. TLC was performed as per the protocol described by Narasimha Rao et al. (2008). The extracted sample (8–10 µL) was spotted on a TLC plate (Merck silica gel 60 F_254_, 20 × 20 cm, normal phase). Ten µL of each trichothecene (1 mg/mL) standard including deoxynivalenol (DON), nivelenol (NIV), T2, and zearalenone (ZEA) from the stock solutions were also spotted on TLC plate. Separation was carried out in solvent system of chloroform–methanol (97:3) used as mobile phase. After separation, the plates were dried in hot air oven at 100 °C for 10 min. The spots were compared with the standards and retention factor (Rf) values for each toxin was calculated.

## Results and discussion

Fungal contamination of cereals with trichothecene producing *Fusarium* spp. is a global problem and it has been reported from different parts of the world. Scientific concern has been bestowed to understand the diversity, incidence and management of trichothecene producing *Fusarium* spp. in wheat and other cereals. However, data on diversity of trichothecene producing *Fusarium* spp. on cereal grains are very limited in Karnataka, India. In order to collect more information on this, a total of 198 samples were collected covering different districts of Karnataka. Mycological examination of 53 maize, 54 sorghum, 37 paddy and 54 wheat samples, revealed the occurrence of 10 different trichothecene producing *Fusarium* spp. including *F. avenaceum*, *F. crookwellense*, *F. culmorum*, *F. equiseti*, *F. graminearum*, *F. nivale*, *F. poae*, *F. sambucinum* and *F. sporotrichioides*. However, the other three *Fusarium* species reported in the present study such as *F. verticillioides*, *F. proliferatum* and *F. anthophilum* are known as the potential producer of fumonisins (Sreenivasa et al. 2008). Occurrence of these species varied between cereals with respect to their diversity and differences were observed in percent incidence, frequency and relative density.

**Fig. 1:**
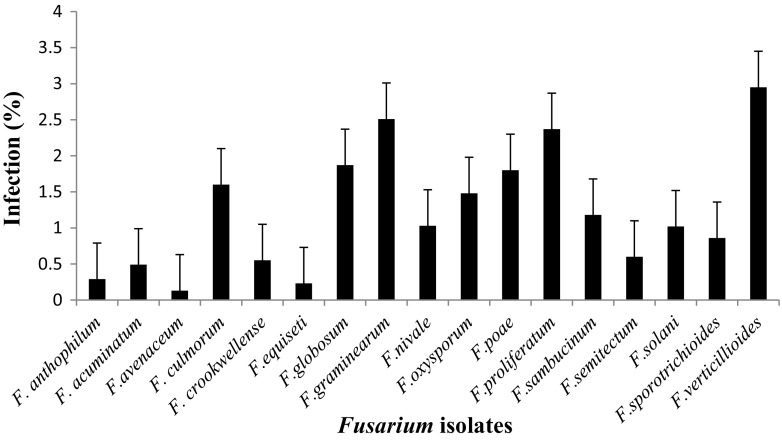
Percent infection of *Fusarium* spp. on maize samples collected from different districts of Karnataka state

**Fig. 2:**
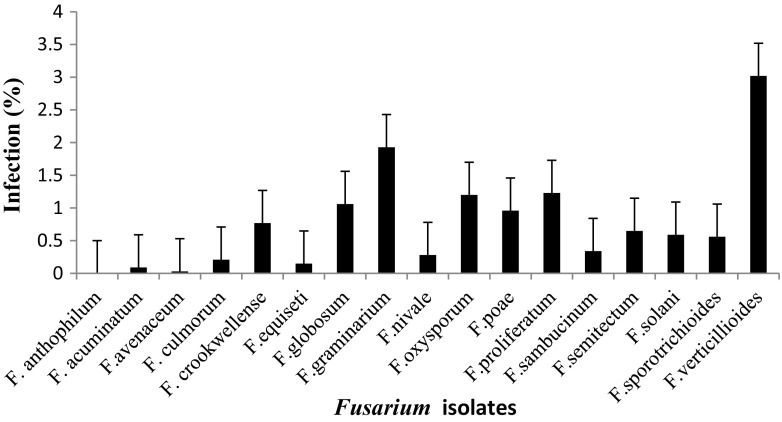
Percent infection of *Fusarium* spp. on sorghum samples collected from different districts of Karnataka state

**Fig. 3:**
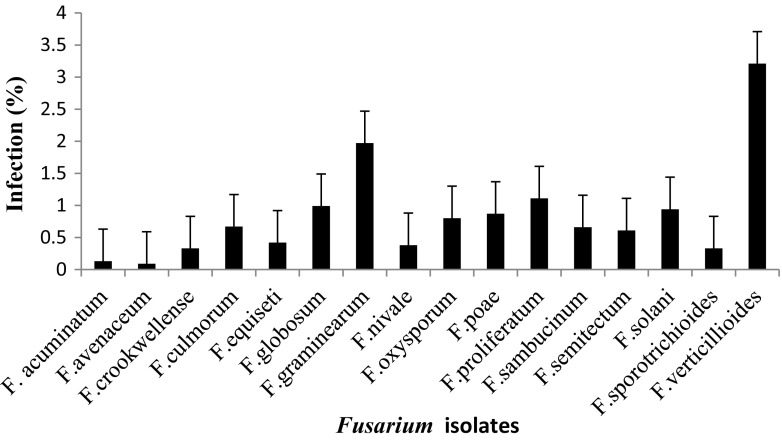
Percent infection of *Fusarium* spp. on paddy samples collected from different districts of Karnataka state

**Fig. 4:**
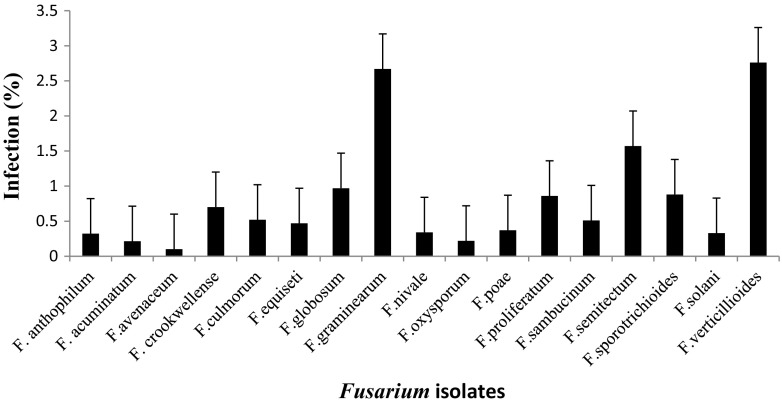
Percent infection of *Fusarium* spp. on wheat samples collected from different districts of Karnataka state

Mycological analysis of different cereal samples by agar plating using MGA.2.5 agar revealed that in maize the highest incidence was *F. verticillioides* with (2.95 %), followed by *F. graminearum* (2.51 %), and *F. proliferatum* (2.37 %) and the least was *F. avenaceum* with incidence of (0.1 %) (Fig. 1). Similarly, percent incidence in sorghum revealed the highest PI was *F. verticillioides* with (3.02 %), followed by *F. graminarium* (1.93 %) and the lowest was *F. avenaceum* (0.03 %) (Fig. 2). In paddy the highest PI was *F. verticillioides* (3.21 %), followed by *F. graminearum* (1.97 %) and *F. prolifaratum* (1.11 %) and least was *F. avenaceum* (0.09 %) (Fig. 3). Percent incidence for wheat revealed that the highest was *F. verticillioides* (2.76 %), followed by *F. graminearum* (2.67 %), with least being *F. avenaceum* (0.10 %) (Fig. 4). *Fusarium* species in different cereals has been reported from different countries. El-Maghraby et al. (1995) isolated four species of *Fusarium* from white hybrids of corn in Egypt. Seventeen species of *Fusarium* were isolated from cereal grains; however, the ability to synthesize mycotoxins limited to 17 species, including the pathogenic ones (Parry et al.^1995^). Gonzalez et al. (1997) reported isolation and occurrence of 1304 *Fusarium* isolates from sorghum grains. Morales et al. (2007) reported the biodiversity in seven *Fusarium* species of Mexico associated with ear rot of maize. Occurrence of 70 *Fusarium* species on different hosts with different geographical locations was also recorded by Leslie and Summerell (2006).

Frequency calculation was done to know the number of samples infected by *Fusarium* among the total number of samples analyzed and accordingly the higher frequency in the maize sample recorded was for *F. graminearum* (79.24 %), and *F. verticillioides* (77.35 %) and the least was for *F. acminatum* (3.77 %). In sorghum higher frequency recorded was for *F. verticillioides* (72.22 %), and *F. oxysporum* (66.66 %) and the least was *F. avenaceum* (1.85 %). Higher frequency for paddy samples was shown by *F. verticillioides* (75.67 %), and *F. sporotrichioides* (62.16 %) and lowest frequency was exhibited by *F. anthophilum*, *F. acuminatum* and *F. avenaceum* with the similar values of (8.10 %). The frequency data for wheat revealed higher values for *F. graminearum* (79.62 %), and *F. verticillioides* (66.6 %) and least was for *F. avenaceum* (3.70 %) (Table 1).

**Table 1:** Frequency and relative density of the *Fusarium* spp. isolated from all the cereal samples collected from different districts of Karnataka state

Sl. no.	*Fusarium* species	Cereal samples							
		Maize		Sorghum		Paddy		Wheat	
		Fr	RD	Fr	RD	Fr	RD	Fr	RD
1	*F. anthophilum*	7.54	1.43	11.11	1.64	8.10	0.88	7.40	1.60
2	*F. acuminatum*	3.77	0.40	5.55	0.71	8.10	1.18	7.40	1.60
3	*F. avenaceum*	11.32	1.71	1.85	0.28	8.10	0.59	3.70	0.87
4	*F. crookwellense*	16.98	2.11	3.70	1.14	10.81	2.29	16.66	5.40
5	*F. culmorum*	30.1	7.89	11.11	6.37	21.62	6.36	14.90	4.52
6	*F. equiseti*	30.1	1.14	9.25	1.07	32.43	3.47	20.37	4.08
7	*F. globosum*	67.9	8.24	33.33	9.31	56.75	7.32	33.33	7.37
8	*F. graminearum*	79.24	13.16	44.44	14.39	51.35	14.43	79.62	22.04
9	*F. nivale*	64.1	5.0	20.37	1.93	56.75	2.29	11.11	2.26
10	*F. oxysporum*	52.83	7.32	66.66	9.02	48.64	5.32	11.11	1.67
11	*F. poea*	60.37	9.61	38.88	7.37	54.05	6.51	29.62	2.91
12	*F. proliferatum*	73.5	10.53	61.11	10.31	59.45	10.65	38.88	6.93
13	*F. sambucinum*	52.8	4.06	22.22	2.72	45.94	2.81	31.48	4.01
14	*F. sporotrichioides*	73.5	4.12	16.66	2.29	62.16	2.36	59.25	6.78
15	*F. semitectum*	32.0	3.0	29.62	4.58	43.24	4.73	48.14	5.98
16	*F. solani*	49.01	4.9	42.5	4.36	56.75	4.51	5.55	0.72
17	*F. verticillioides*	77.35	15.16	72.22	2.79	75.67	23.16	66.66	21.16

*Fr* frequency, *RD* relative density

The relative density was also analyzed for the *Fusarium* species screened. The higher relative density observed in maize was for *F. verticillioides* (15.16 %), *F. graminearum* (13.16 %), *F. proliferatum* (10.53 %) and the lowest density observed was for *F. acuminatum* (0.40 %). Similarly in sorghum the highest relative density recorded was *F. graminearum* (14.39 %) and the least was *F. acuminatum* with (0.71 %). The relative density of *Fusarium* isolates for paddy showed the higher with *F. verticillioides* (23.16 %), *F. graminearum* (14.43 %), and *F. proliferatum* (10.65 %) and the lowest was with *F. avenaceum* (0.59 %). Similarly in wheat the relative density of *Fusarium* species revealed the highest occurrence for *F. graminearum* (22.04 %) and the lowest for *F. avenaceum* (0.87 %) (Table 1). Similar data were obtained in the State of Parana, Brazil, where they recorded high percent incidence of *Fusarium* species on cereals (Ono et al. 1999). Ghiasian et al. (2004) reported predominance of species of *Fusarium* (38.5 %) among all other fungi studied. Dass et al. (2007) reported high incidence of *F. verticillioides* in maize and sorghum-based animal feed stuffs and poultry feed mixtures produced in Karnataka, India. Worldwide, approximately 25 % of crops are affected by mycotoxigenic *Fusarium* species annually. The most notorious among the toxigenic *Fusarium* species include *F. verticillioides*, *F. graminearum*, *F. sporotrichioides*, *F. proliferatum* and *F. acuminatum.* In the present study, all these important toxigenic *Fusarium* spp. showed their association with cereal grains in Karnataka state. Most of these are known mycotoxin producers. The naturally occurring *Fusarium* mycotoxins belong to trichothecenes, zearalenones and fumonisins groups. Moreover, moniliformin, beauvericin and fusaproliferin have also been found in naturally infected cereals and are considered as emerging toxicological problems.

**Fig. 5:**
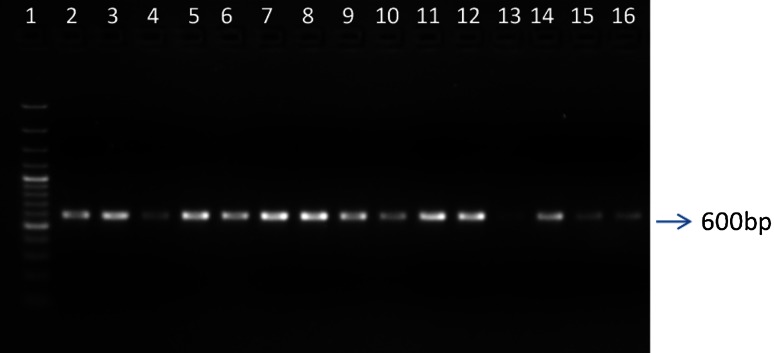
Agarose gel of *Fusarium* spp. amplified by using ITS set of primers: *lane 1* marker of 1000 kb, *lane 2* *F. acuminatum*, *lane 3* *F. sporotrichioides*, *lane 4* *F. equiseti*, *lane 5* *F. venenatum*, *lane 6* *F. poae*, *lane 7* *F. graminearum*, *lane 8* *F. avenacium*, *lane 9* *F. sambucinum*, *lane 10* *F. nivale*, *lane 11* *F. crookwellense*, *lane 12* *F. anthophilum*, *lane 13* *F. semitectum*, *lane 14* *F. arminacium*, *lane 15* and *16* *F. culmorum*

All the 14 representatives of different *Fusarium* isolates were quantified by PCR analysis using ITS primers and all the samples were positive for the ITS regions and expected amplicon size was 600 bp (Fig. 5). Identification of the individual *Fusarium* species was made on the basis of ITS gene differences. Identification of fungal species by using phenotypic characteristics is confusing; this is particularly complex in case of genus *Fusarium* because of the existence of several and often-conflicting taxonomic treatments. Molecular detection tools have been used to detect contamination by *Fusarium* species in cereals (Sreenivasa et al. 2008; Abd-El-Salam et al. 2003). In view of this, in the present investigation PCR with sequencing technique was used to detect *Fusarium* species associated and this serves as secondary confirmation to the microscopic based identification.

**Fig. 6:**
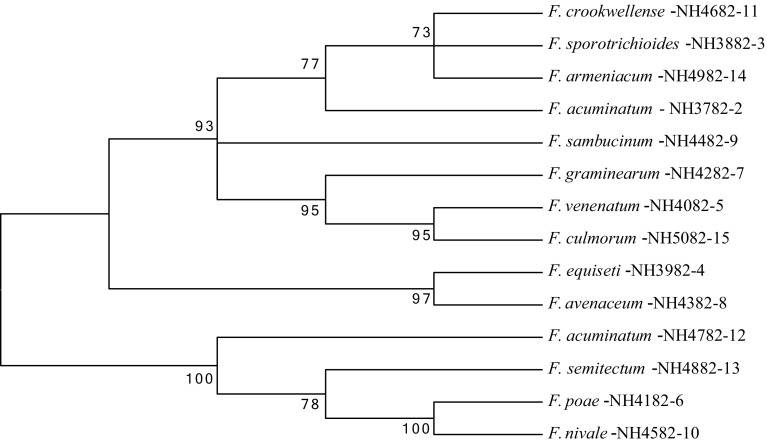
Phylogenetic tree constructed for the *Fusarium* spp. isolated from cereals

Further, the PCR amplified products were subjected to sequence analysis and the obtained readings were compared with the available data base at NCBI. Results revealed 96–100 % similarities for all the *Fusarium* species with E value being 0. The sequence readings obtained for all the *Fusarium* species were also used for the construction of phylogenetic tree to know the evolutionary relationships among the *Fusarium* species based on their genetic closeness. In our studies it has shown that the interpretation of the phylogenetic tree exhibited three major congruent with well-resolved major clades with potent trichothecene producing *Fusarium* species in all the three major clades showing significantly >70 % boot strap values (Fig. 6) which are considered as positive values for determining the genetic similarity within the species. Combined analysis of 14 *Fusarium* species was done for the similarity clusters with external nodes indicates with NCBI accession numbers (Table 2), for individual *Fusarium* species and branches reflected the nodes from the major clade. The potent trichothecene producers like *F. crookwellense*, *F. sporotrichioides*, *F. graminearum* and *F. culmorum* were from same evolutionary origin from the same major clade revealed that they are genetically similar with not much significant differences.

**Table 2:** NCBI accession numbers obtained after the sequence submission of *Fusarium* spp

Sl. no.	*Fusarium* species	NCBI Accession number
1	*F. acuminatum*	KJ371100
2	*F. sporotrichioides*	KJ371098
3	*F. equiseti*	KJ371094
4	*F. venenatum*	KJ371103
5	*F. poae*	KJ371096
6	*F. graminearum*	KJ371099
7	*F. avenacium*	KJ371102
8	*F. sambucinum*	KJ371095
9	*F. nivale*	KJ371097
10	*F. crookwellense*	KJ371105
11	*F. anthophilum*	KJ371093
12	*F. semitectum*	KJ371106
13	*F. arminacium*	KJ371101
14	*F. culmorum*	KJ371104

**Fig. 7:**
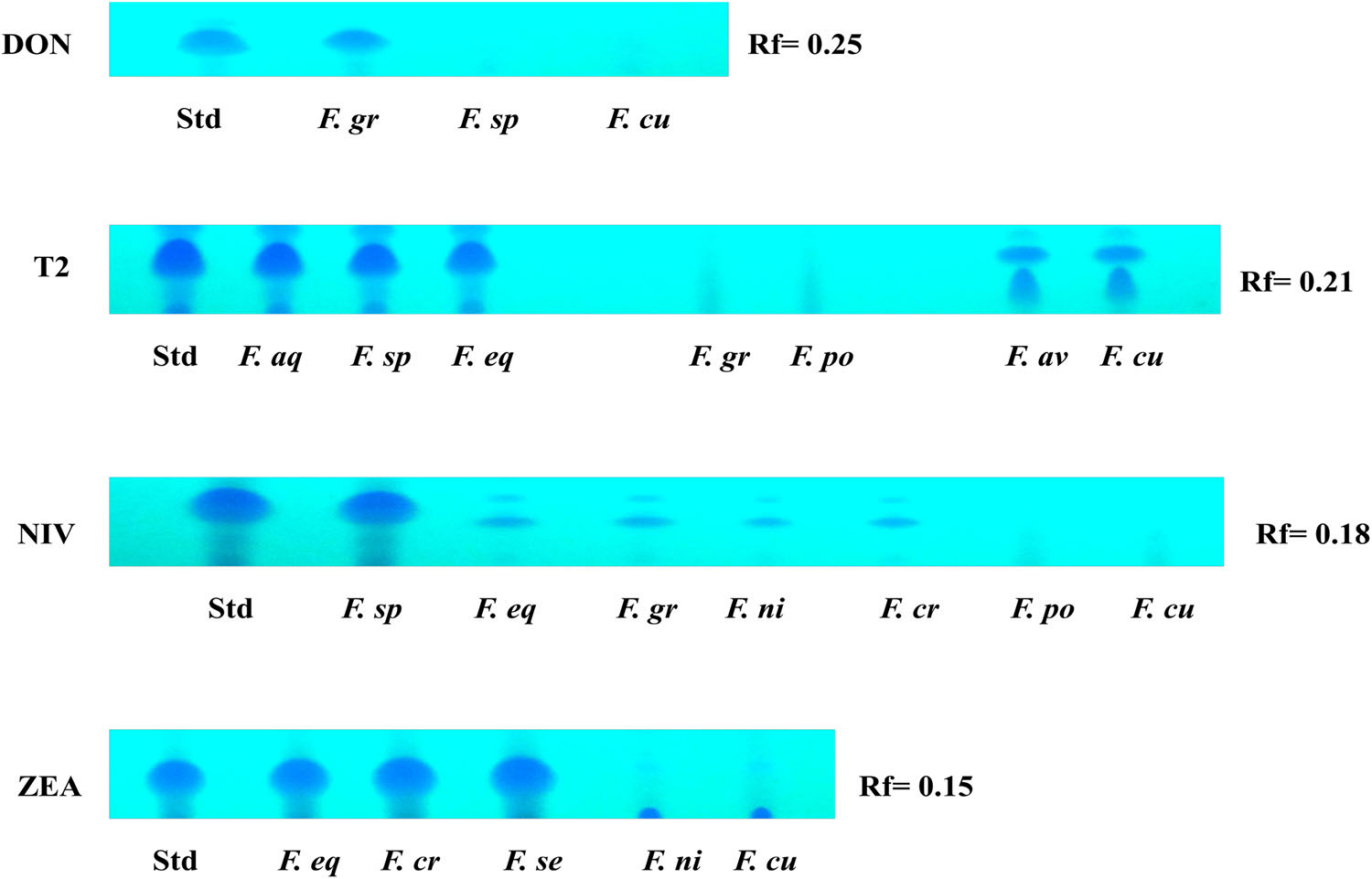
Thin layer chromatographic analysis of trichothecenes production by *Fusarium* spp. Std, standard toxin; F. gr, *Fusarium graminearum*; F. sp, *F. sporotrichioides*; F. cu, *F. culmorum*; F. aq, *F. acuminatum*; F. eq, *F. equiseti*; F. po, *F. poae*; F. av, *F. avenaceum*; F. ni, *F. nivale*; F. cr, *F. crookwellense*; F. se, *F. sambucinum*

TLC studies revealed that the ability of *Fusarium* species to produce trichothecenes. All ten *Fusarium* species were positive for the production of trichothecenes such as T2, DON, NIV and ZEA. *F. graminarium* was found to be positive for DON production with the Rf value 0.25 and *F. acuminatum*, *F. sporotrichioides*, *F. equesiti*, *F. avenaceum and F. culmorum* were positive for production of T-2 for which the Rf value is 0.21. The production of nivalenol with Rf value of 0.18 was observed in *F. nivale*, *F. graminarium*, *F. equiseti* and *F. crookwellense*. Similarly *F. sporotrichioides*, *F. equeseti*, *F. graminearum*, *F. crookwellense* and *F. semitectum* were also positive for the production of zearalenone with the Rf value of 0.15 (Fig. 7). Trichothecenes constitute a large group of *Fusarium* mycotoxins which are members of sesquiterpenoids. These toxins are classified as type A and type B based on their chemical structure. T-2, HT-2, deoxynivalenol (DON), nivalenol (NIV) and zearalenone (ZEA) are some of the common trichothecenes found in cereals (Desjardins 2006). Considered as most toxic and potent inhibitors of protein synthesis, they possess immunosuppressive and cytotoxic effects (Canady et al. 2001). They are produced mainly by *F. sporotrichioides*, *F. poae*, *F. equiseti*, *F. acuminatum*, and *F. graminearum* in cereal and cereal-based products. The natural occurrence of DON, NIV and ZAE in cereals in around 19 countries was surveyed and reported by Toshitsugu et al. (1998).

The extent of percent incidence, frequency and relative density of trichothecene producing *Fusarium* spp. indicated the extent of contamination and seed damage with respect to physiological and biochemical quality parameters of cereals. Growth of *Fusarium* species on cereals can reduce the germination along with the loss of carbohydrate, protein and oil content increases the moisture content, free fatty acid and thus reduces the dry matter content. The growth also causes discoloration of grain, heating, mustiness, dry matter loss, and production of several secondary metabolites such as mycotoxins, which are potentially dangerous to humans and animals (Bhattacharya and Raha 2002). The data on the incidence and diversity of *Fusarium* species on cereals would be of great value for this region for predicting the extent of post-harvest infection, colonization and subsequent deterioration of cereal grains.
